# High Fasting Glycemia Predicts Impairment of Cardiac Autonomic Control in Adults With Type 2 Diabetes: A Case-Control Study

**DOI:** 10.3389/fendo.2021.760292

**Published:** 2021-11-09

**Authors:** Lucas Raphael Bento Silva, Paulo Gentil, Camila Simões Seguro, Gabriela Teles de Oliveira, Maria Sebastiana Silva, Antônio Roberto Zamunér, Thomas Beltrame, Ana Cristina Silva Rebelo

**Affiliations:** ^1^ Department of Physical Education, Araguaia University Center, Goiânia, Brazil; ^2^ Post-Graduate Program in Health Sciences, Faculty of Medicine, Federal University of Goias, Goiânia, Brazil; ^3^ Faculty of Physical Education and Dance, Federal University of Goias, Goiânia, Brazil; ^4^ Hypertension League, Federal University of Goias, Goiânia, Brazil; ^5^ Laboratory of Clinical Research in Kinesiology, Department of Kinesiology, Universidad Católica del Maule, Talca, Chile; ^6^ Institute of Computing, University of Campinas, São Paulo, Brazil; ^7^ Department of Physical Therapy, Federal University of São Carlos, São Paulo, Brazil; ^8^ Department of Morphology, Institute of Biological Sciences, Federal University of Goias, Goiânia, Brazil

**Keywords:** type 2 diabetes, physical exercise, autonomic modulation, fasting glycemia, heart rate variability, heart rate recovery

## Abstract

**Introduction:**

Type 2 diabetes (T2D) is characterized by a metabolic disorder that elevates blood glucose concentration. Chronic hyperglycemia has been associated with several complications in patients with T2D, one of which is cardiac autonomic dysfunction that can be assessed from heart rate variability (HRV) and heart rate recovery (HRR) response, both associated with many aspects of health and fitness, including severe cardiovascular outcomes.

**Objective:**

To evaluate the effects of T2D on cardiac autonomic modulation by means of HRV and HRR measurements.

**Materials and Methods:**

This study has an observational with case-control characteristic and involved ninety-three middle-aged adults stratified into two groups (control group - CG, n = 34; diabetes group - DG, n = 59). After signing the free and informed consent form, the patients were submitted to the evaluation protocols, performed biochemical tests to confirm the diagnosis of T2D, collection of R-R intervals for HRV analysis and cardiopulmonary effort test to quantify HRR.

**Results:**

At rest, the DG showed a reduction in global HRV (SDNN= 19.31 ± 11.72 *vs* CG 43.09 ± 12.74, p < 0.0001), lower parasympathetic modulation (RMSSD= 20.49 ± 14.68 *vs* 52.41 ± 19.50, PNN50 = 4.76 ± 10.53 *vs* 31.24 ± 19.24, 2VD%= 19.97 ± 10.30 *vs* 28.81 ± 9.77, p < 0.0001 for both indices) and higher HRrest when compared to CG. After interruption of physical exercise, a slowed heart rate response was observed in the DG when compared to the CG. Finally, a simple linear regression showed that fasting glycemia was able to predict cardiac autonomic involvement in volunteers with T2D.

**Conclusion:**

Patients with T2D presented lower parasympathetic modulation at rest and slowed HRR after physical exercise, which may be associated with higher cardiovascular risks. The findings show the glycemic profile as an important predictor of impaired cardiac autonomic modulation.

## Introduction

Diabetes mellitus affects about 463 million people worldwide with 32 million cases in South and Central America. According to the Diabetes Atlas of the International Diabetes Federation (IDF) in 2019, it is estimated that more than 4.2 million people died due to the disease and its complications and type 2 diabetes mellitus (T2D) accounted for 90-95% of diagnoses (1)

T2D is usually caused by the association of deficiency in insulin release by β cells located in the pancreas with the inefficiency of tissues in responding to insulin (2), which results in impaired glucose metabolism and hyperglycemia (3, 4). Most T2D also have high blood glucose, which is associated with a higher risk of all-cause mortality, especially due to severe cardiovascular events (5). In addition, T2D has been associated with deleterious effects on nerve fibers that make up the autonomic nervous system (ANS) (6, 7), which might be associated with further health risks.

In light of ANS damage and impairted heart functionality, heart rate variability (HRV) has become an important tool for assessing autonomic impairment in people with T2D (8). Previous studies have shown through linear models of analysis reduction of HRV in T2D patients when compared to healthy individuals (9–11) and hyperglycemia was a predictor for cardiac autonomic modulation dysfunction (12). Another possibility of HRV analysis is by the nonlinear model, which assesses the complexity of the HRV signal in different populations (13, 14); however, its use in studies with T2D patients is scarce.

Heart rate recovery (HRR) has also been described as an important marker of impaired cardiac autonomic function (15). A slowed HRR response implies a higher risk of all-cause mortality in the general population (16). In T2D patients, its delay is associated with elevated blood glucose levels (17). Such findings may reinforce the suggestion that chronic changes in serum blood glucose levels may cause damage to the cardiovascular system (18, 19), and highlight HRV analyses as an option for assessing cardiovascular impairment in diabetic patients. However, there are few studies that evaluated HRV by linear and nonlinear models and HRR in an additional way at rest and in response to stress, to better understand the effects of T2D on cardiac autonomic control (11, 17, 20).

Thus, the aim of this study was to evaluate cardiac autonomic control of T2D patients through linear and nonlinear measurements of HRV and HRR. The hypothesis is that T2D patients have negative changes in autonomic modulation (reduction in parasympathetic modulation, increase in sympathetic modulation and lower HRV) and that there is an association between blood glucose levels and such changes.

## Materials and Methods

### Study Design

This study has observational case-control characteristics. The sample consisted of two groups: cases, those individuals with confirmed diagnosis of T2D (DG), and control, those healthy individuals with no history of previous diseases (CG). The DG was composed of individuals recruited in an event held by the Banco de Olhos de Goiás Foundation in partnership with the Diabetes League of Federal University of Goias (UFG), entitled “3rd Diabetes Task Force”, while the CG was selected from a previous database with healthy individuals according to the pairing criteria.

The present study had a pairing in the 2:1 model, in which each control was matched by age and level of self-reported physical activity. Recruitment followed the following order: for every two cases inserted in the study, one case was selected.

The inclusion criteria for DG were: a) patients with T2D aged 45-65 years; b) fasting glycemia above 126mg/dL; c) glycated hemoglobin equal to or greater than 6.4%; (d) who had not participated in any exercise programme in the last six months. For the CG, the inclusion criteria were: a) healthy patients with no history of cardiometabolic diseases aged 45-65 years; b) that met the criteria of pairing by age, BMI and level of physical activity.

**Figure 1:**
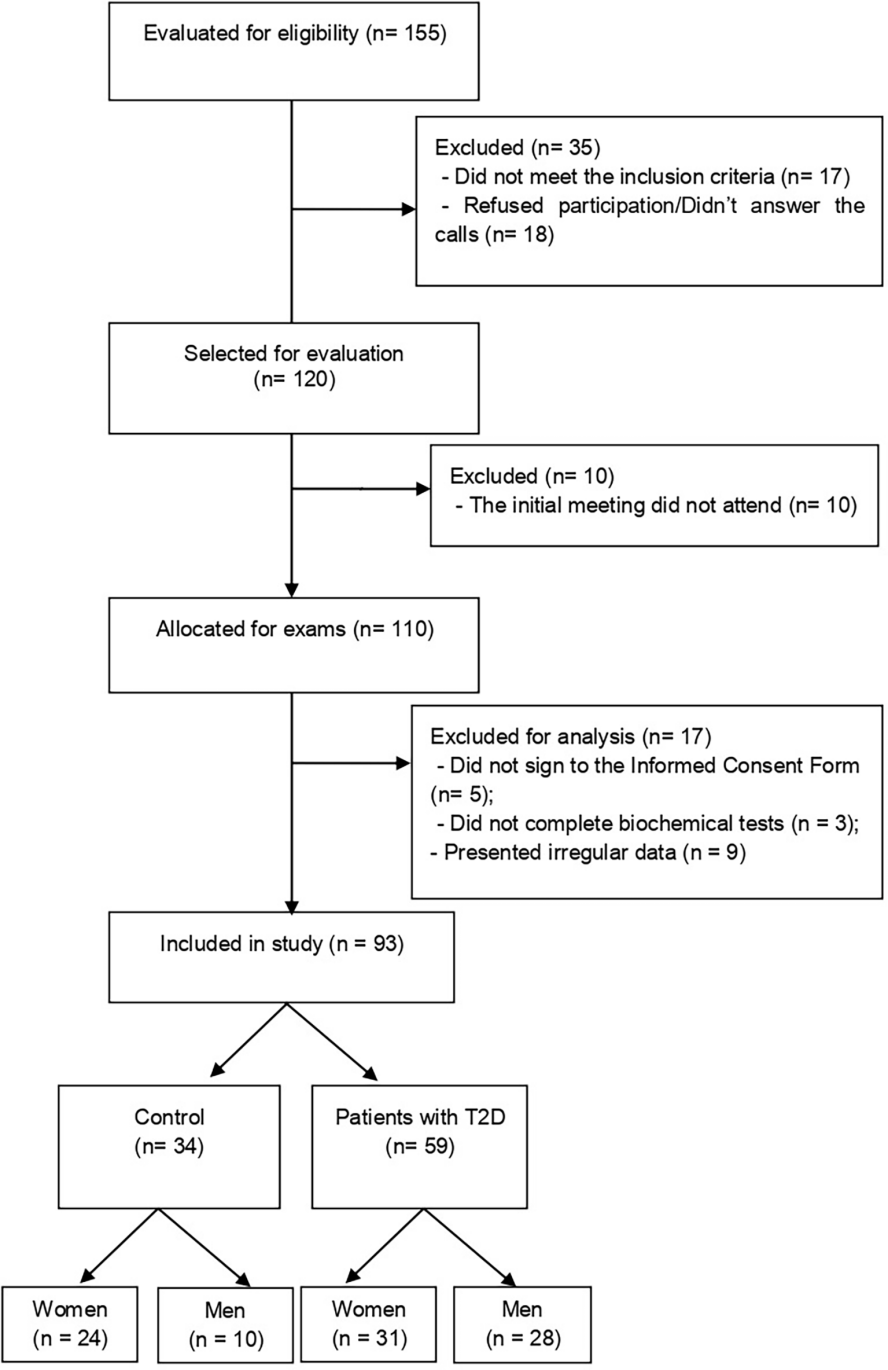
Diagram of the selection flow and inclusion of study participants. T2D, type 2 diabetes.

The exclusion criteria for DG were: self-reported infectious diseases in any of the baseline evaluation stages, arrhythmias and/or frequent extrasystoles under rest conditions or triggered during physical exertion, unstable angina and previous history of acute myocardial infarction, chronic obstructive pulmonary disease or kidney disease, osteomyoarticular alterations and neurological deficit that prevented the understanding of any of the stages of the study. The flowchart of this study is shown in **Figure 1**.

The study was approved by the Research Ethics Committee of the institution under opinion no. 2.667.732 and followed all the recommendations of the national responsible bodies. The participants were informed about the objectives and methods used and those who consented participated in the study.

### Evaluation Protocols

All evaluations were previously scheduled with the participants, and took place in the morning (from 8:00 a.m. to 12:00 a.m.), in an air-conditioned room with relative humidity between 40 and 60% and temperature between 22 and 24°C, according to recommendations of the American College of Sports Medicine – ACSM (21). The volunteers received guidance so that on all evaluation days they would not drink alcohol and/or stimulants, not to perform intense physical exercises in the 24 hours prior to the evaluations, to have a moderate meal at least two hours before the execution of the tests (except for the day of biochemical evaluation in which they should be fasting for 12 hours) and maintain the medications used.

All were familiar with the evaluation room and the equipment used before the beginning of each protocol. Moments before the tests, participants answered an anamnesis about their health status, time of T2D diagnosis, use of medications, practice of physical activity and compliance with previous recommendations. They were instructed not to speak unnecessarily during evaluations to avoid interference in the capture of signals and to report any change in the general state before, during and after protocols to the researchers.

#### Glycemic Profile

Blood collection was performed on a different day of the other evaluations after 12-hour fasting. The samples were collected by the vacuum method in 4mL EDTA tubes (Plastilab, São José dos Pinhais - PR) and transported in thermal suitcases under temperature control between 2 and 8°C.

Homogenized whole blood was used for preparation and processing. Fasting glycemia was measured by the colorimetric enzymatic method and the diagnosis followed the reference criteria of the American Diabetes Association *(ADA)* (22).

#### Heart Rate Variability (HRV)

For HRV evaluation, volunteers were instructed to remain at rest for approximately ten minutes to stabilize the hemodynamic variables in recording the R-R interval (iR-R). Then, the signal was recorded at rest, in the supine position, for six minutes, by a cardiofrequencymeter (Polar v800, Finland) fixed in the chest and with simultaneous transmission to a clock. Data were stored and transferred to specific software (PolarFlow, Finland) for proper analysis and downloaded to a portable microcomputer. The HRV indices obtained were analyzed using Kubios HRV software (version 3.2).

In the time domain analyses, the SDNN indices (standard deviation of all normal RR intervals recorded in a time interval, expressed in ms) were used as measurement of the overall HRV, RMSSD (square root of the mean of the differences between normal intervals of adjacent RR in time intervals expressed in ms) was used as a marker of parasympathetic modulation. PNN50, which represents the percentage of R-R intervals with a variation greater than 50 milliseconds, also corresponds to the vagal modulation.

Symbolic analysis and Shannon Entropy were also performed, the first was performed by grouping all possible symbolic patterns into four families: (I) equal patterns, without the presence of any variation (0V, as an example: 2-2-2 or 4-4-4 or 5-5-5), (II) patterns with a variation (1V, as an example of patterns: 2-2-3 or 4-2-2), (III) two similar variations forming an ascending or descending line (2LV, i.e. 5-3-1 or 1-2-4) and (IV) patterns with two different variations, in which the three symbols form the representation of peak or valley (2UV, such as 1-4-2 or 5-2-4), being evaluated by the frequency of occurrence (0V%, 1V%, 2VS% and 2VD%). A previous study evaluated that the 0V family represents sympathetic modulation, the 1V pattern reflects vagal and sympathetic modulations, while 2LV also reflects parasympathetic and sympathetic modulations with vagal predominance and, finally, the family with 2UV standard represents, exclusively, parasympathetic modulation (13)

Shannon Entropy (ES), which reflects the complexity of the distribution of these symbolic patterns, is considered low if the distribution is flat (patterns have been equally distributed) and high when a subset of patterns is unlikely, absent, or rarely (23).

#### Cardiopulmonary Exercise Test (CPET)

The evaluation of cardiorespiratory fitness was performed directly through the CPET. The load increment protocol applied was ramp type, with total test time from 8 to 12 minutes. Each volunteer started the test with a two minutes warm-up and adaptation, starting with the velocity and inclination corresponding to 50% of the initial values predicted for age and sex (24). Treadmill velocity was increased during warm up by 0.5 km/h every 15 seconds. Cool down lasted four minutes, with 20% velocity reduction every 20 seconds. The ergometer used was the treadmill (Micromed^®^, Centurion 200, Brasília, Brazil) coupled to a computer for data processing.

The treadmill was chosen because it presented greater similarity with the pattern of walking movement. During the execution of the CPET protocol, HR, blood pressure (SBP and DBP) data were collected. HR was continuously monitored by cardiac monitor (Polar v800, Finland). BP records were obtained by Korotkoff’s auscultatory method, using a mercury column sphygmomanometer (WanMed, São Paulo, SP, Brazil) and a stethoscope (Littman, St. Paul, MN, USA). The criteria for interruption of The CPET were those recommended by the ACSM (21): 1) loss of stride on the treadmill; 2) obtaining the maximum heart rate (HRmax) predicted for the participant’s age and 3) respiratory exchange ratio ≥1,15 (gas analysis was performed by the Cortex Metalyzer II analyzer, Leipzig, Germany).

#### Resting Heart Rate

Resting heart rate (HRrest) was measured using a cardiofrequency meter (Polar, v800, Finland). The participants remained at rest for ten minutes to stabilize the hemodynamic variables and then data collection was performed during the six-minute period in the supine position. Data were obtained from the formula: HR = 60000/iR-R.

#### Heart Rate Recovery (HRR)

HRR is a measure that expresses HR decay after interruption of an intense physical exercise. The HR for this analysis was obtained during the performance of the CPET, the volunteers were monitored by a cardiofrequency meter during and for four minutes after the interruption of the test in the standing position while still on the treadmill.

The HRR was calculated from the difference between the peak HR during physical exercise, the first minute (HRR-1min) and second minute of recovery (HRR-2min). The HRR data were also obtained after the interruption of the CPET, filtered and analyzed through its own routine developed in the OriginPro 8.0 software (OriginLab, Northampton, MA, USA), which applies an exponential model to the data referring to the entire recovery period (four minutes of cooldown and three minutes of seated rest) (25, 26).

To obtain the best parameters of this exponential curve, a nonlinear algorithm that uses the minimization of the sum of squared errors as a convergence criterion was used. Only the r function > 0.95 the final analyses was added. The HRR was modulated according to time, as presented in the formula:


(1) $$\text{HR}(\text{t})=\text{HRpeak}-{\text{a}}^{*}(1–{\text{e}}^{-\left(t–\text{TD}\right)/\text{t}})$$

where the constant “t” is time (offTAU); “HRpeak” is the peak HR during CPET; “Amp” (a*) is the amplitude of HR decay after the end of the exercise (offAMP); and “TD” is the time delay for the function. The inclusion of the term “TD” in this function was established due to the possibility of HRR not being immediately reduced after the interruption of the load. Since the parameter “τ” is a time constant in a decreasing negative exponential function, it can be inferred that the lower its value, the faster the HRR kinetics (27).

### Statistical Analysis

Data normality was tested using the Shapiro-Wilk test, variance homogeneity was evaluated using the Levene test and boxplot graphs were used to identify significant outliers. T-test was used to compare cardiac autonomic modulation variables between groups. Pearson’s correlation was used for choosing variables to be added in the simple linear regression, those variables that presented correlation coefficient above 0.20 (r = 0.20) were added. To verify the relationship of prediction between fasting glucose and HRV indices, simple linear regression was used, for the composition of the statistical model fasting glucose was used as independent variables and the following variables were used as dependent factors: iR-R, HRrest, SDNN, RMSSD, 0V%, 2VS%, 2VD%, SE, HRR1, HRR2, offAMP, offTAU. Statistical analysis was performed using the statistical program Statistical Package for the Social Sciences (SPSS; Armonk, NY; IBM Corp.), version 21. p < 0.05 was considered significant in all analyses.

## Results

**Table 1** show the general characteristics of the volunteers. Comparative analysis between groups showed a significant difference in all variables analyzed, except for age (p = 0.089) and that the individuals in the DG presented higher mean values for weight, height, BMI, SBP and DBP.

**Table 1:** Characterization of the individuals evaluated.

	CG (n = 34)	DG (n = 59)	p-value
**Features**			
Age (years)	53.38 ± 8.43	56.10 ± 9.15	.089
Weight (kg)	71.32 ± 13.23	79.03 ± 15.78	**.022**
Height (m)	1.59 ± 0.44	1.64 ± 0.09	**.001**
BMI (kg/m²)	27.97 ± 4.95	30.02 ± 1.57	**.025**
SBP (mmHg)	124.29 ± 15.76	135.10 ± 18.58	**.005**
DBP (mmHg)	79.47 ± 10.02	87.17 ± 11.93	**.004**
Time of diabetes (years)	–	10.42 ± 6.96	
Blood glucose (mg/dL)	86.32 ± 9.80	145.20 ± 59.59	**<.001**
HbA1c (%)	5.59 ± 0.60	8.97 ± 2.05	**<.001**
***Cardiovascular risk factors***			
Hypertension	2 (5,88%)	33 (55%)	–
Obesity	3 (8,82%)	22 (36.66%)	–
Smoking	–	1 (1.66%)	–
***Drugs***			
*Hypoglycemic Drugs*	–	43 (71.66%)	–
Biguanides	–	38 (63.33%)	
Sulphonylureas	–	11 (18.33%)	
SGLT2 inhibitor	–	1 (1.66%)	
*Insulin*	–	3 (5%)	–
*Antihypertensive Drugs*	–	27 (45%)	–
Diuretics	–	15 (25%)	
Beta-blockers	–	–	
Angiotensin II antagonists		19 (31.66%)	

BMI, body mass index; SBP, systolic blood pressure; DBP, diastolic blood pressure; SGLT-2, sodium-glucose cotransporter 2; HbA1c, hemoglobin glycated. **Bold values**, statistically considered between the two groups.

As expected, fasting glucose (p < 0.0001) were significantly higher in patients with T2D. **Table 1** also shows the presence of cardiovascular risk factors and the use of medications to control T2D.

**Table 2** describes HRV indices. The results show impaired cardiac autonomic modulation in patients with T2D with lower values in the following variables: iR-R, HRpeak, SDNN, RMSSD, PNN50, 2VS% and 2VD%. In the DG, higher values were observed in the variables HRrest and 0V%. There was no significant difference for the variables 1V% and Shannon entropy.

**Table 2:** Comparison of heart rate variability indices.

Variables			
	CG (n=34)	DG (n=59)	p-value
R-Ri, ms	875.03 ± 106.30	839.41 ± 144.54	**<.0001**
HRrest, bpm	69.33 ± 7.65	73.53 ± 12.57	**.048**
HRpeak, bpm	172.97 ± 15.07	149.47 ± 15.85	**<.0001**
Heart Rate Variability			
Linear Analysis			
SDNN, ms	43.09 ± 12.74	19.31 ± 11.72	**<.0001**
RMSSD, ms	52.41 ± 19.50	20.49 ± 14.68	**<.0001**
PNN50, %	31.24 ± 19.24	4.76 ± 10.53	**<.0001**
Symbolic Analysis			
0V%	20.41 ± 15.69	31.49 ± 18.73	**.002**
1V%	38.45 ± 11.10	38.85 ± 11.53	.435
2VS%	12.10 ± 8.66	9.37 ± 6.54	**.046**
2VD%	28.81 ± 9.77	19.97 ± 10.30	**<.0001**
Shannon Entropy	3.62 ± 0.71	3.35 ± 0.88	.059

HRrest, resting heart rate, supine position; HRpeak, heart rate at the peak of physical exercise; R-Ri, R-R intervals; SDDN, standard deviation of all normal RR intervals recorded in a time interval, expressed in milliseconds; RMSSD, square root of the mean square differences between adjacent normal RR intervals, in a time interval, expressed in milliseconds; PNN50, percentage of adjacent iR-R with duration differences greater than 50 ms; 0V%, percentage of the pattern without variation; 1V%, percentage of the pattern with a variation; 2VS%, percentage of the pattern with two similar variations; 2VD%, percentage of the pattern with two different variations. **Bold values**, statistically considered between the two groups.

**Figure 2:**
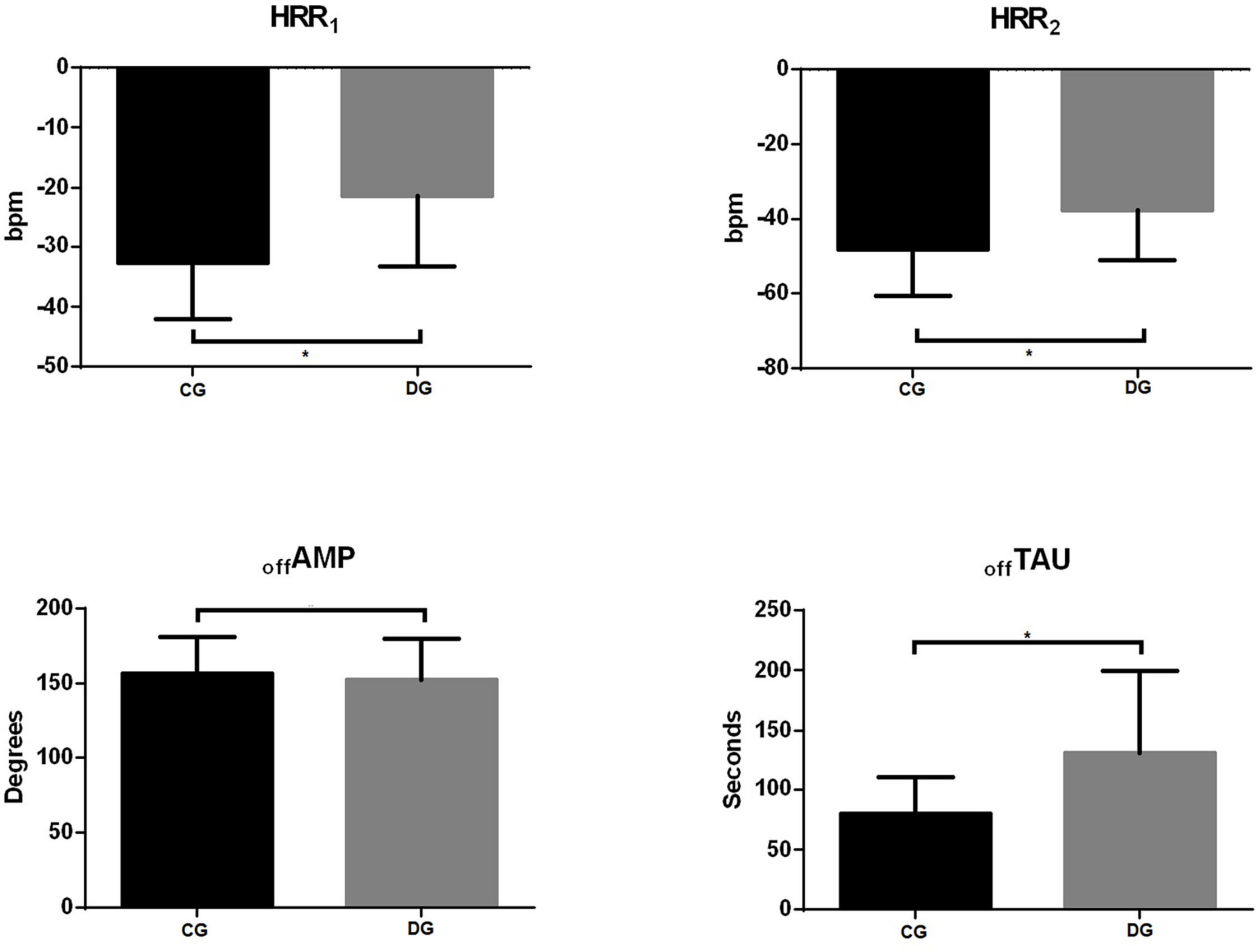
Heart rate recovery behavior. HRR1, number of beats in the 1st minute after interruption of physical exercise; HRR2, number of beats in the 2nd minute after interruption of physical exercise; offAMP, amplitude of the heart rate decay curve; offTAU, time constant. *Statistically significant (p < 0.05).

**Figure 2** shows the behavior of HRrest were a statistical difference was observed between groups for HRR1, HRR2 and offTAU (p < 0.0001), evidencing the slowness in the HR response for participants with T2D after interruption of physical exercise.

**Figure 3:**
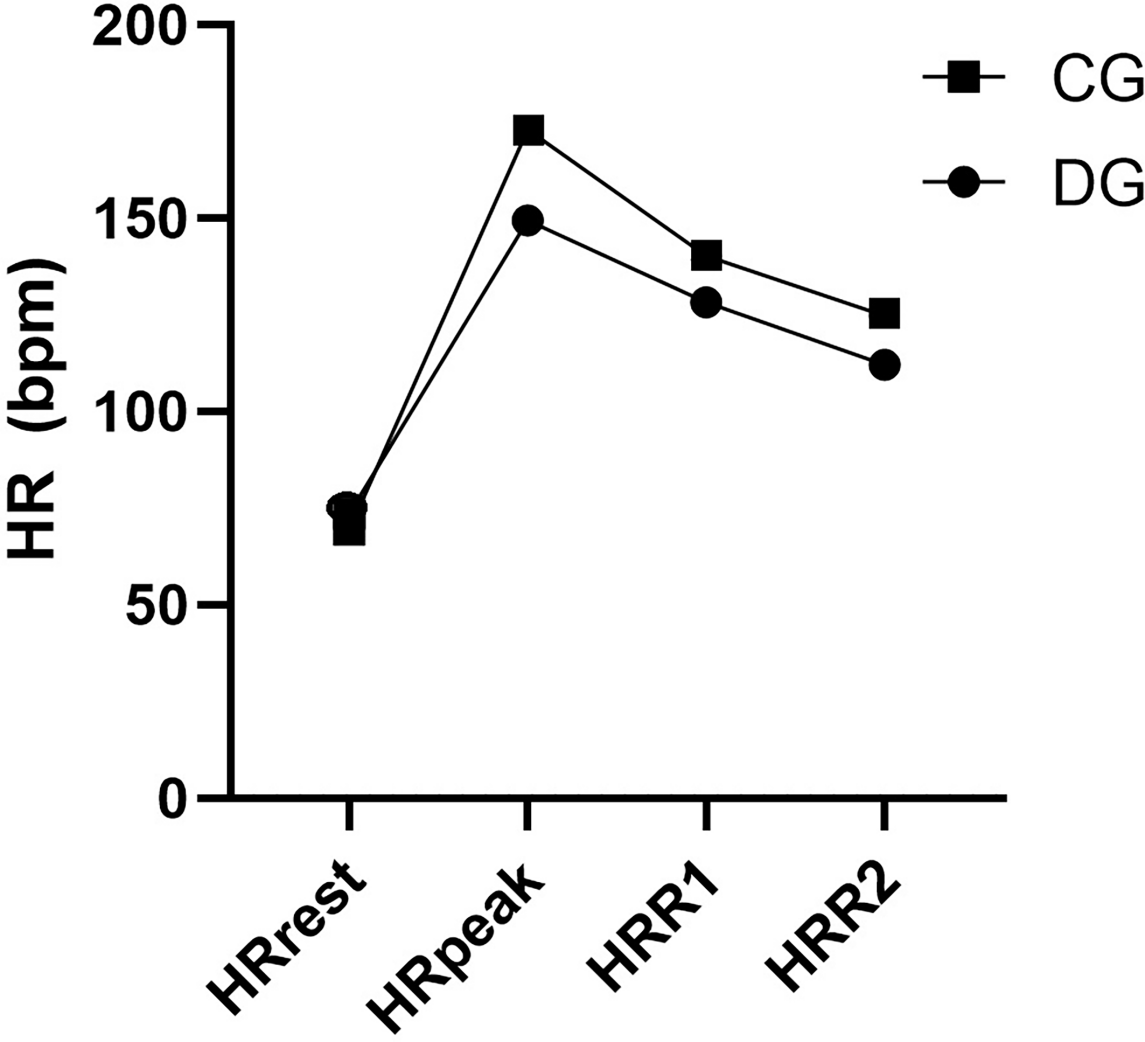
Comparison of heart rate behavior in the groups evaluated. HR presented in bpm. HRrest, resting heart rate; HRpeak, heart rate during peak exertion; HRR1min, heart rate after the first minute of recovery; HRR2min, heart rate after the first second of recovery.

**Figure 3** shows us a comparison between the mean values of HR behavior between groups, one can perceive the greater slowing of HR in the DG, evidencing the impairment of cardiac autonomic control in T2D.

**Table 3** shows whether fasting glycemia is associated with HRV and HRR analysis. Fasting glycemia in DG was negatively associated with the following variables: iR-R, SDNN, RMSSD and PNN50 (p = 0.035, 0.010, 0.010 and 0.41, respectively) and positively associated with HRrest, HRR1 and HRR2 (p = 0.010, 0.003 and 0.032, respectively). In CG, no associations were found between HRV and HRR analysis and fasting glycemia.

**Table 3:** Simple linear regression between heart rate kinetics and variability and fasting glycemia.

Variable	FG		
	β adjusted	CI 95%	p-value
R-Ri	-.348	-1.664 – -.061	**.035**
HRrest	.421	.023 –.160	**.010**
HRV			
SDNN	-.426	-.150 – -.021	**.010**
RMSSD	-.424	-.188 – -.026	**.010**
0V%	-.079	-.133 –.082	.637
2VS%	-.041	-.043 –.033	.808
2VD%	.138	-.035 –.084	.417
Entropia de Shannon	.122	-.003 –.007	.467
HRR			
HRR1	.470	-.033 –.158	**.003**
HRR2	.355	.008 –.159	**.032**
_off_AMP	.151	-.064 –.170	.367
_off_TMR	-.059	-.501 –.352	.728

HRrest, resting heart rate, supine position; HRR, heart rate at the peak of physical exercise; R-Ri, R-R intervals; SDDN, standard deviation of all normal RR intervals recorded in a time interval, expressed in milliseconds; RMSSD, square root of the mean square differences between adjacent normal RR intervals, in a time interval, expressed in milliseconds; 0V%, percentage of the pattern without variation; 2VS%, percentage of the pattern with two similar variations; 2VD%, percentage of the pattern with two different variations. HRR1, number of beats in the 1st minute after interruption of physical exercise; HRR2, number of beats in the 2nd minute after interruption of physical exercise; offAMP, amplitude of the heart rate decay curve; offTAU, constant t time. Bold values, statistically considered between the two groups.

## Discussion

The aim of this study was to evaluate the influence of T2D on cardiac autonomic modulation in the resting condition through linear and nonlinear analyses, and after peak physical exercise, through monoexponential analysis of HRR.

The results showed that T2D patients presented impairments in cardiac autonomic modulation evidenced by linear (SDNN, RMSSD and PNN50) and symbolic analyses of HRV (symbolic analysis and Shannon entropy) at rest. A slowing of the HRR was also observed after interruption of physical exercise when compared to CG and fasting glycemia was associated with HRV values.

HRrest is associated with a higher risk of severe cardiovascular outcomes in T2D patients, such as: myocardial infarction, stroke and all-cause mortality (5). It is believed that the increase of one unit in the standard deviation HRrest results in a 20% increase in the risk of complications. Moreover, the increase in HRrest implies a state of chronic excitation of several tissues and when combined with hyperglycemia it might activate specific pathways that result in neuronal ischemia and cellular degeneration (28, 29). In our sample, higher values of this variable were observed in individuals with T2D, and fasting glycemia was associated with this increase, reinforcing the importance of glycemic control and measures that minimize such impairments.

Our findings corroborate the results of Verma et al. (20), that compared individuals with and without T2D and observed lower HRV (SDNN, RMSSD and PNN50 indexes) in T2D patients. Some studies have shown that in several diseases there is a reduction in parasympathetic modulation and an increase in sympathetic modulation (20, 30). This is seen in obese patients with metabolic syndrome and can be explained by increased central adiposity, activation of the hypothalamic-pituitary-adrenal (HPA) axis by chronic stress and decreased baroreflex sensitivity (31). Shah et al. (32), when evaluating young individuals with T2D and comparing with obese individuals without T2D, found that T2D presented lower value for SDNN, RMSSD and PNN50, evidencing that metabolic dysfunction caused greater impairment of cardiac autonomic modulation in the sample studied.

This study is one of the few that performed the symbolic analysis in individuals with T2D. It is observed that the DG presented higher sympathetic modulation found in the variable 0V%, and lower parasympathetic modulation that can be observed in the variable 2VD% when compared to the CG. These data are in accordance with what was observed by Moura-Tonello et al. (6) when comparing men with and without T2D under postural change in which they found higher values of sympathetic modulation (represented by the variable 0V%), and lower balance between sympathetic and vagal modulations from the variable 2VS%. The results also show that the participants of the present study presented lower significant values in parasympathetic modulation (represented by the variable 2VD%). Autonomic dysfunction is one of the main complications of T2D, and is associated with a mortality risk five times higher than in other individuals (33).

Previous study from our group showed attenuation of HRR response in patients with metabolic syndrome (12). This study also verified an association between blood glucose levels and the slowed HRR response in women who presented the risk factors for metabolic syndrome.

In relation to the HRR analysis, the study by Sacre et al. (34) establishes ideal cutoff points for HRR values in patients with T2D. In the first minute after physical exercise HR should decrease ≥ 28bpm and in the second minute ≥ 50 bpm. In the present study, it was observed that the DG presented lower values of the variables HRR1 and HRR2 when compared to the CG, and these values are below (HRR1: -25.11 ± 11.02 and HRR2: -40.11 ± 13.08) those established by the aforementioned authors. Confirming such data, Verma et al. (20) also found slowing in HRR after interruption of physical exercise when comparing individuals with and without T2D after an incremental effort test.

Once physical exertion begins, mechanisms from the spinal cord cardiovascular control center activate arterial baroreflex by stimulating a “rapid vagal withdrawal” as the intensity of physical exercise increases, the greater the action of the baroreflex and muscle metaboreceptors that promote greater vagal withdrawal and increase sympathetic modulation, and this increases according to the increase in intensity by the action of the simpatoadrenal system. After the interruption of the exercise, HR recovery occurs through the integration between the rapid sympathetic withdrawal and the beginning of vagal resumption, which has been shown to be an important marker of adverse events (35). This is in line with the present results that show the slowing of the HRR response, evidencing those T2D patients present impairment in cardiac autonomic control.

Michael et al. (36, 37) showed that HR response after exercise is dependent on exercise intensity and muscle metaboreflex seems to moderate the cardiac autonomic response after physical exercise. As exercise intensities exceed the 2nd ventilatory threshold, there is an exacerbated increase in circulating catecholamines and activation of beta-adrenergic receptors that play an important role in sympathetic modulation which, in turn, can promote a slowed HRR after cessation of physical exercise. Thus, it can be inferred that physiological responses are dependent on the intensity of the stimulus. However, the mechanisms that may explain the attenuation in HRR in patients with T2D still need further elucidation.

The present results suggest that T2D impacts cardiac autonomic control and these effects are associated with elevated serum glucose levels. Even in patients with good glycemic control by pharmacological measures, structural and functional changes in the heart muscle are already installed (38–40).

In the diabetic heart, chronic hyperglycemia might result in changes in myocardial rhythm and contractility, impairing cardiac performance (41). Due to cellular toxicity caused by high levels of blood glucose in cellular layers, cardiomyocytes demonstrate increased oxidative stress (generation of reactive oxygen species – ROS) that initiates the release of the poly enzyme (ADP-ribose) polymerase-1 (PARP) which can result in cellular damage (40) associated with systolic and diastolic dysfunction.

In addition, there is an increase in the generation of advanced glycation products (AGEs) and these form bonds with other macromolecules such as the binding with collagen that results in interstitial fibrosis and the binding with the enzymes SERCA-2 and RyR, responsible for the release of Ca2+ by the sarcoplasmic reticulum (38, 39). These changes promote contractility and relaxation of cardiomyocytes. Finally, these subcellular changes may promote hyperactivity of the reinnine-angiotensin-aldosterone and sympathetic nervous systems (40, 42).

Thus, it is believed that after the installation of T2D it is interesting to find alternatives to improve cardiovascular function. In this regard, physical exercise would be an alternative to minimize the effects of T2D, since it can improve systolic and diastolic function. However, it is necessary to be carefully prescribed and autonomic impairment should be evaluated by simple and specific tools, as the used in this study.

### Study Limitations

One important limitation of the study is the absence of glycated hemoglobin analysis in our biochemical tests, which could provide important information about the impact of long term glycemia.. Future prospective studies should include this and also evaluate the effects of physical exercise as a non-pharmacological measure for controlling autonomic impairment in T2D.

## Conclusion

Patients with type 2 diabetes presented impairment in cardiac autonomic modulation with greater sympathetic modulation and lower vagal modulation when compared to healthy individuals at rest and in response to physical exercise. Fasting glycemia was associated with cardiac autonomic dysfunction.

## Data Availability Statement

The raw data supporting the conclusions of this article will be made available by the authors, without undue reservation.

## Ethics Statement

The studies involving human participants were reviewed and approved by Research Ethics Committee of the Federal University of Goias. The patients/participants provided their written informed consent to participate in this study.

## Author Contributions

LS and AR: concept and study design, collected and analyzed data, wrote, reviewed, and edited the manuscript. CS and GO: collected data and edited the manuscript. PG, MS, AZ, TB, and AR: contributed with data analysis, reviewed, and edited the manuscript. All authors contributed to the article and approved the submitted version.

## Conflict of Interest

The authors declare that the research was conducted in the absence of any commercial or financial relationships that could be construed as a potential conflict of interest.

## Publisher’s Note

All claims expressed in this article are solely those of the authors and do not necessarily represent those of their affiliated organizations, or those of the publisher, the editors and the reviewers. Any product that may be evaluated in this article, or claim that may be made by its manufacturer, is not guaranteed or endorsed by the publisher.
